# Rapid processing of threatening faces in the amygdala of nonhuman primates: subcortical inputs and dual roles

**DOI:** 10.1093/cercor/bhac109

**Published:** 2022-03-22

**Authors:** Mikio Inagaki, Ken-ichi Inoue, Soshi Tanabe, Kei Kimura, Masahiko Takada, Ichiro Fujita

**Affiliations:** Laboratory for Cognitive Neuroscience, Graduate School of Frontier Biosciences, Osaka University, 1-4 Yamadaoka, Suita, Osaka 565-0871, Japan; Center for Information and Neural Networks, National Institute of Information and Communications Technology and Osaka University, 1-4 Yamadaoka, Suita, Osaka 565-0871, Japan; Systems Neuroscience Section, Primate Research Institute, Kyoto University, 41-2 Kanrin, Inuyama, Aichi 484-8506, Japan; Systems Neuroscience Section, Primate Research Institute, Kyoto University, 41-2 Kanrin, Inuyama, Aichi 484-8506, Japan; Systems Neuroscience Section, Primate Research Institute, Kyoto University, 41-2 Kanrin, Inuyama, Aichi 484-8506, Japan; Systems Neuroscience Section, Primate Research Institute, Kyoto University, 41-2 Kanrin, Inuyama, Aichi 484-8506, Japan; Laboratory for Cognitive Neuroscience, Graduate School of Frontier Biosciences, Osaka University, 1-4 Yamadaoka, Suita, Osaka 565-0871, Japan; Center for Information and Neural Networks, National Institute of Information and Communications Technology and Osaka University, 1-4 Yamadaoka, Suita, Osaka 565-0871, Japan

**Keywords:** multisynaptic route, pulvinar, rabies virus, superior colliculus, temporal visual cortex

## Abstract

A subcortical pathway through the superior colliculus and pulvinar has been proposed to provide the amygdala with rapid but coarse visual information about emotional faces. However, evidence for short-latency, facial expression-discriminating responses from individual amygdala neurons is lacking; even if such a response exists, how it might contribute to stimulus detection is unclear. Also, no definitive anatomical evidence is available for the assumed pathway. Here we showed that ensemble responses of amygdala neurons in monkeys carried robust information about open-mouthed, presumably threatening, faces within 50 ms after stimulus onset. This short-latency signal was not found in the visual cortex, suggesting a subcortical origin. Temporal analysis revealed that the early response contained excitatory and suppressive components. The excitatory component may be useful for sending rapid signals downstream, while the sharpening of the rising phase of later-arriving inputs (presumably from the cortex) by the suppressive component might improve the processing of facial expressions over time. Injection of a retrograde trans-synaptic tracer into the amygdala revealed presumed monosynaptic labeling in the pulvinar and disynaptic labeling in the superior colliculus, including the retinorecipient layers. We suggest that the early amygdala responses originating from the colliculo–pulvino–amygdalar pathway play dual roles in threat detection.

## Introduction

The amygdala is critical for detecting potential danger through sensory inputs (Klüver and Bucy 1939; LeDoux 2000). Many neurons in the primate amygdala respond to a particular facial expression, for instance a threatening appearance, which is a cue for potential danger (Leonard et al. 1985; Nakamura et al. 1992; Kuraoka and Nakamura 2006; Gothard et al. 2007). The anterior part of the temporal visual cortex, a major source of visual inputs to the amygdala (Aggleton et al. 1980; Amaral et al. 1992; Cheng et al. 1997; Stefanacci and Amaral 2000), also contains neurons that respond to emotional faces (Hasselmo et al. 1989; Nakamura et al. 1994; Sugase et al. 1999). Selective responses to emotional faces are found in specific subregions of the temporal cortex such as the fundus of the superior temporal sulcus (Hadj-Bouziane et al. 2008; Zhu et al. 2013; Taubert et al. 2020). Further upstream in the ventral cortical pathway, multiple clusters of face-responsive neurons (face patches) process facial information (Rolls 2000; Tsao et al. 2006, 2008; Freiwald and Tsao 2010). In addition to the ventral cortical pathway, a subcortical pathway through the superior colliculus and then the pulvinar has been proposed to provide the amygdala with rapid, but coarse, visual information about facial expressions (the “dual-route hypothesis”; Morris et al. 2001; Johnson 2005; Pegna et al. 2005; Tamietto and de Gelder 2010). This notion originates from studies on rodents (LeDoux 2000) and has been extended to human and nonhuman primates. The subcortical pathway is suggested to underlie rapid, subconscious, coarse detection of threat signals such as fearful faces. The dual-route hypothesis can help explain some of the symptoms of a variety of neurological/developmental disorders, including hemineglect, blindsight, and autistic spectrum disorders, as well as face recognition by newborns (Johnson 2005; Tamietto and de Gelder 2010; but see Buiatti et al. 2019 for cortical responses to face-like patterns in newborns). Several studies in patients provide evidence for the dual-route system in the human visual system (de Gelder et al. 1999; Pegna et al. 2005).

However, the supporting evidence is insufficient in many ways (Pessoa and Adolphs 2010). Critically, evidence for short-latency, expression-discriminating responses from individual neurons is nonexistent, despite a number of studies in both the human and monkey amygdala (e.g. Kuraoka and Nakamura 2006; Gothard et al. 2007; Mormann et al. 2008; Rutishauser et al. 2011). Recent electrophysiological recordings in the human amygdala demonstrated that intracranial local field potentials (LFPs) discriminate fearful faces from other faces within 74 ms after stimulus onset (Méndez-Bértolo et al. 2016). The discrepancy between single-neuron and LFP results remains unresolved. Moreover, even if this rapid response exists at the single-neuron level, how it contributes to the detection of facial expressions remains unknown.

In addition, anatomical evidence for the subcortical route to the amygdala is inconclusive. The medial pulvinar receives projections from the deeper layers of the superior colliculus (Benevento and Fallon 1975; Benevento and Standage 1983) and sends projections to the lateral nucleus of the amygdala (Jones and Burton 1976; Norita and Kawamura 1980; Romanski et al. 1997), but the connections within the medial pulvinar remain unclear. A recent study revealed superior colliculus axons running near medial pulvinar neurons that project to the amygdala (Elorette et al. 2018). However, evidence is still lacking for multisynaptic connections to the amygdala from the superior colliculus, especially from its superficial layers that receive projections from retinal ganglion cells (Pollack and Hickey 1979).

Here, we addressed these unresolved issues by analyzing responses of single neurons in the monkey amygdala to threatening, neutral, and affiliative facial expressions. We demonstrated that ensemble responses of amygdala neurons carry robust information about threatening faces within 50 ms after stimulus onset. This early response contained excitatory and suppressive components. The excitatory responses were beneficial for transferring rapid signals to downstream areas. The suppressive responses highlighted the onset of later, sustained, excitatory inputs, presumably from the temporal cortex. Furthermore, by examining spatial frequency (SF) tuning properties, we obtained evidence that early excitation is mediated by subcortical processing, whereas the suppressed neurons receive slow inputs from the temporal visual cortex. We thus suggest that these amygdala responses play dual roles in threat detection, i.e. rapid transfer of threat signals downstream to behavioral/autonomic response stages and elaboration of late-arriving cortical inputs over time. Finally, we provide anatomical evidence for the shortest route from the retina to the amygdala through the superior colliculus and pulvinar. Our findings may define a mechanism whereby threatening faces are rapidly detected via the colliculo–pulvino–amygdalar pathway.

## Materials and methods

### Care and use of animals

Single-unit recording was conducted at Osaka University. All animal care and experimental procedures were approved by the Animal Experiment Committee of Osaka University in compliance with the National Institutes of Health “Guide for the Care and Use of Laboratory Animals” (Revised in 1996). Two adult Japanese monkeys (*Macaca fuscata*; monkey SA, male, 9.0 kg; monkey KU, female, 7.0 kg) were used for the physiological experiments. Tracer experiments were performed at the Primate Research Institute (PRI) of Kyoto University. The experimental protocols were approved by the Ethics Committee of the PRI. All experiments were conducted in accordance with the *Guide for Care and Use of Laboratory Primates* by the PRI. Two adult rhesus monkeys (*Macaca mulatta*; monkey HE, 6.5 kg, female; monkey SE, 9.6 kg, male) were used for the anatomical experiments.

## Single-neuron recording in the amygdala and temporal visual cortex

### Surgery for single-unit recording

Monkeys were premedicated with atropine sulfate (0.03 mg/kg, i.m.) and sedated with ketamine hydrochloride (5 mg/kg, i.m.). All surgical procedures were performed under anesthesia with isoflurane (1%–3% in 70% N_2_O and 30% O_2_) and aseptic conditions. Local anesthesia was applied with lidocaine (2%) as needed. Arterial oxygen saturation level, body temperature, heart rate, and electrocardiogram were continuously monitored. A head holder and recording chamber were attached to each monkey, and their positions were determined with the aid of magnetic resonance images, which were taken at the National Institute for Physiological Sciences. The chamber was centered at 20 or 21 mm anterior and 10 mm lateral to the ear canals with a 10° lateral tilt relative to the midline. Postoperatively, the monkeys were treated with an antibiotic (piperacillin sodium, 40 mg/kg, i.m.), an anti-inflammatory/analgesic agent (diclofenac sodium, 1 mg/kg suppository; or ketoprofen, 0.8 mg/kg i.m.), and a corticosteroid (dexamethasone, 0.1 mg/kg i.m.) for 1 week. After a recovery period (more than 2 weeks), we started to train the monkeys in a fixation task (see below).

### Visual stimuli and task

**Figure 1:**
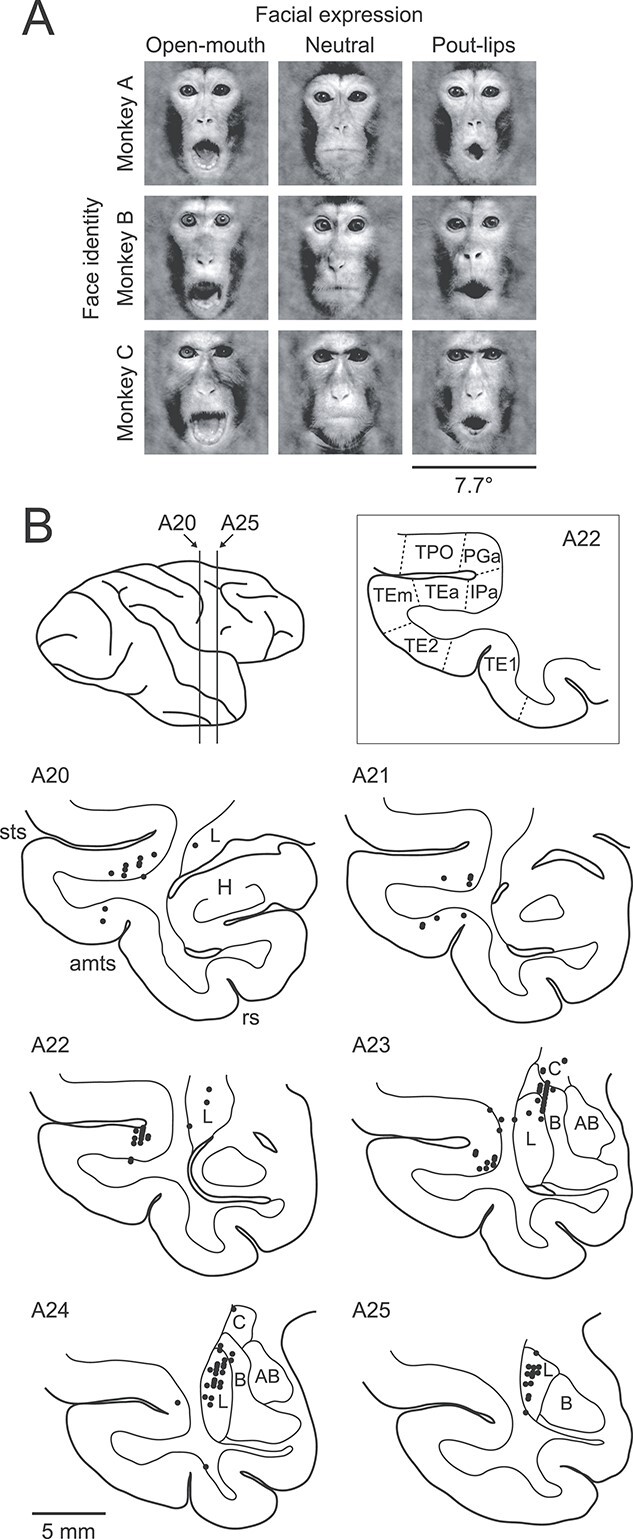
Visual stimuli and recording sites. A) The stimulus set consisted of 9 face images with 3 different facial expressions (open mouth, neutral, pout-lips) displayed by 3 different monkeys. B) Reconstructed recording sites (A20–A25) in the right hemisphere of monkey SA. H, hippocampus; L, lateral nucleus of the amygdala; B, basal nucleus of the amygdala; AB, accessory basal nucleus of the amygdala; C, central nucleus of the amygdala; sts, superior temporal sulcus; amts, anterior middle temporal sulcus; rs, rhinal sulcus. Cytoarchitectonic areas TPO, PGa, IPa, TEa, TEm, TE2, and TE1 of the temporal cortex are based on Baylis et al. (1987).

Nine images of 3 monkeys, each displaying 3 different facial expressions (open mouth, neutral, pout-lips), were used as face stimuli (Fig. 1A). In the open-mouth expression, the monkeys opened their mouths widely by lowering the jaw and exposing the lower teeth. In the pout-lips expression, the monkeys contracted their mouths into a rounded shape. It has been suggested that open mouth is an aggressive expression whereas pout-lips is an affiliative expression (Hinde and Rowell 1962; Van Hooff 1962, 1967), although interpretation of the meanings of the facial expressions in nonhuman primates remains controversial and challenging (Hadj-Bouziane et al. 2008; Sterck and Goossens 2008; Beisner and McCowan 2014; Waller et al. 2020; Taubert and Japee 2021). The bared-teeth appearance (or fear grin), an effective facial expression to elicit neural responses in the amygdala and temporal cortex (Hadj-Bouziane et al. 2008; Zhu et al. 2013; Liu et al. 2017; Taubert et al. 2020), was not included in our face stimuli. This was because the monkeys subjected to the photo session were accustomed to other monkeys and experimenters and rarely showed the bared teeth reaction. After isolating faces from body features and background scenes, the mean luminance and 2-dimensional amplitude spectrum were equalized across the face images to minimize differences in low-level visual features (Inagaki and Fujita 2011). Visual stimuli were presented on a gamma-corrected CRT monitor (HM903D-A; Iiyama; screen size, 32.8° × 25.5° in visual angle; resolution, 1,600 pixels × 1,200 pixels; refresh rate, 85 Hz) with an OpenGL program running on a computer (Precision 330; Dell). Luminance ranged from 0.02 to 46 cd/m^2^, and the background luminance was 22 cd/m^2^. All face images encompassed 7.7° × 7.7° on the monitor. For each neuron tested, we presented the face images to the monkeys at least 6 times in a pseudo-random order (mean: 9.9 times). Although monkeys were able to expect the timing of the stimulus appearance, they were unable to expect which face image would appear in the upcoming trial.

During recording experiments, the monkeys performed a fixation task while sitting in a primate chair. After the monkeys fixated a small dot (0.18° × 0.18°) at the center of the screen for 500 ms, a face image was presented for 500 ms in that location. The monkeys obtained a liquid reward for maintaining fixation within the fixation window throughout the trial. If the monkeys failed to maintain fixation, the trial was terminated without any reward and the data were discarded. Gaze direction was monitored with an infrared camera system. The size of the fixation window was 2° × 2° for monkey SA and 3.5° × 3.5° for monkey KU.

### Electrophysiology

**Figure 2:**
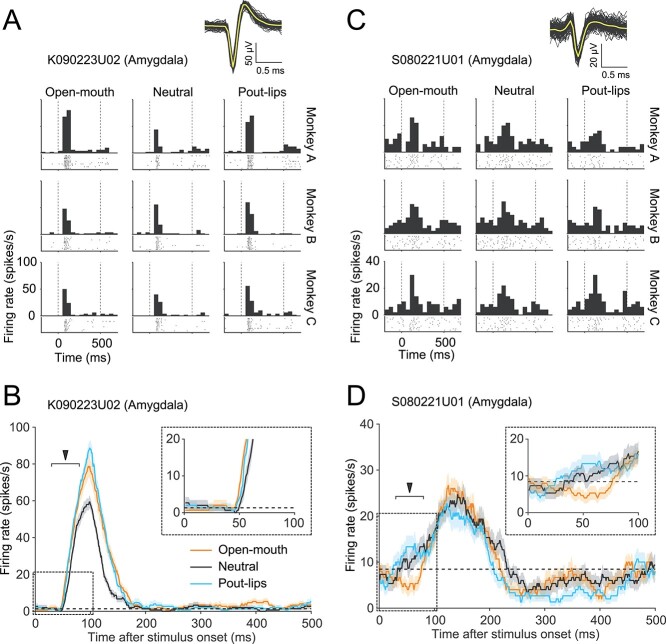
Selectivity for facial expressions in individual neurons in the amygdala. A, C) Spike waveforms, raster plots, and peristimulus time histograms (PSTHs) of 2 example neurons in the amygdala. Waveforms of spikes (*n* = 100) are superimposed for each neuron. Yellow traces are mean waveforms averaged across the spikes. For raster plots and PSTHs, each panel corresponds to responses to each facial image as per the layout of the panels in Fig. 1A. B, D) Time course of responses for different facial expressions (open mouth, orange; neutral, black; pout-lips, cyan) in 2 individual neurons in the amygdala (mean ± SEM, 50-ms sliding window). Dashed lines represent the mean FR immediately before stimulus presentation (−50 to 0 ms). The early window (30–80 ms) is indicated by the filled arrowhead.

Stainless steel guide tubes and tungsten electrodes (0.2–2.0 MΩ at 1 kHz; Frederick-Haer) were used for recording extracellular single-neuron (unit) activity. After penetrating the dura mater with the aid of a guide tube, an electrode was inserted into the brain through the guide tube. The tip of the guide tube was positioned at approximately 10 mm above the recording sites. The voltage signals were amplified (×10,000) and filtered (bandpass: 500 Hz to 3 kHz) by an amplifier (MEG-6116; Nihon Kohden) and stored on a computer (sampling rate: 20 kHz) for off-line spike sorting. All results shown in this paper are based on data from off-line spike sorting; spikes were extracted using the template-matching method and then classified into one or more units based on their amplitude (insets in Fig. 2A and C). For online monitoring, we isolated extracellular action potentials with a spike-sorting system (Multi Spike Detector; Alpha-Omega).

### Statistical analyses

We used nonparametric statistical tests (2-sided Wilcoxon signed-rank test, Friedman test, and Spearman’s rank correlation) to examine the statistical significance of the data. Error bars and shaded areas in Figs. 2B, D and 4D, F denote standard errors of the mean. We generated null distributions of the data by shuffling the stimulus–response relationships (1,000 repetitions) and estimated confidence intervals of the null distributions. We performed all analyses in MATLAB (MathWorks).

### Data analysis for single-unit recording

Face responsiveness was determined by comparing the firing rate (FR) during the 500 ms before stimulus onset with that during the 500 ms after stimulus onset. This analysis was performed separately for each face image. Neurons were considered face responsive if at least 1 of the 9 face images elicited a significant increase in activity (2-sided Wilcoxon signed-rank test, *P* < 0.05).

We first tested the statistical significance of differential responses to the 3 facial expressions (Friedman test, main factor: facial expression) in a 50-ms time window centered at 55 ms after stimulus onset (the “early window”; 30–80 ms). We focused on this specific time window because the shortest response latency of pulvinar neurons to faces and face-like patterns is 30 ms (Nguyen et al. 2013). We counted the number of facial expression-discriminating neurons (criterion: *P* < 0.05) across the population of recorded neurons. To estimate false positives, we generated a null distribution of the number of facial expression-discriminating neurons by shuffling the stimulus–response relationships (1,000 repetitions). This determined the 95th and 99th percentiles of the null distribution, allowing false-positive estimations at statistical criteria of *P* = 0.05 and *P* = 0.01. 

**Figure 3:**
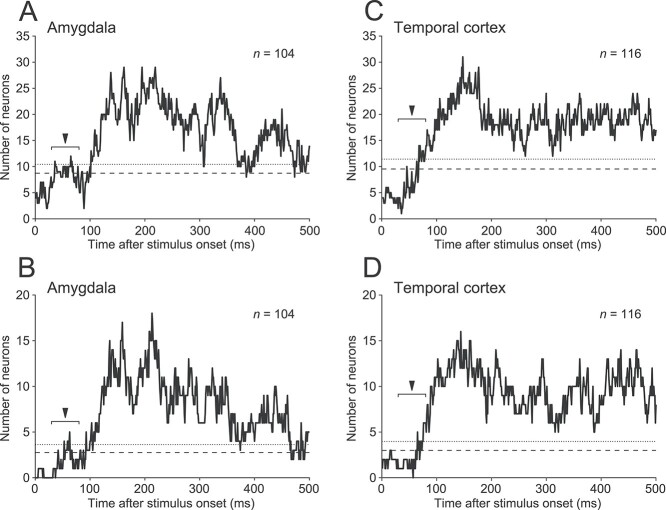
Time course of the number of expression-differentiating neurons (Friedman test, 50-ms sliding window) in the amygdala (A, B) and temporal cortex (C, D). The statistical criteria of the Friedman test were *P* < 0.05 in A and C, and *P* < 0.01 in B and D. Dashed and dotted lines represent the 95th and 99th percentiles of the null distribution generated by shuffling the data, respectively. The early window (30–80 ms) is indicated by the filled arrowhead.

**Figure 4:**
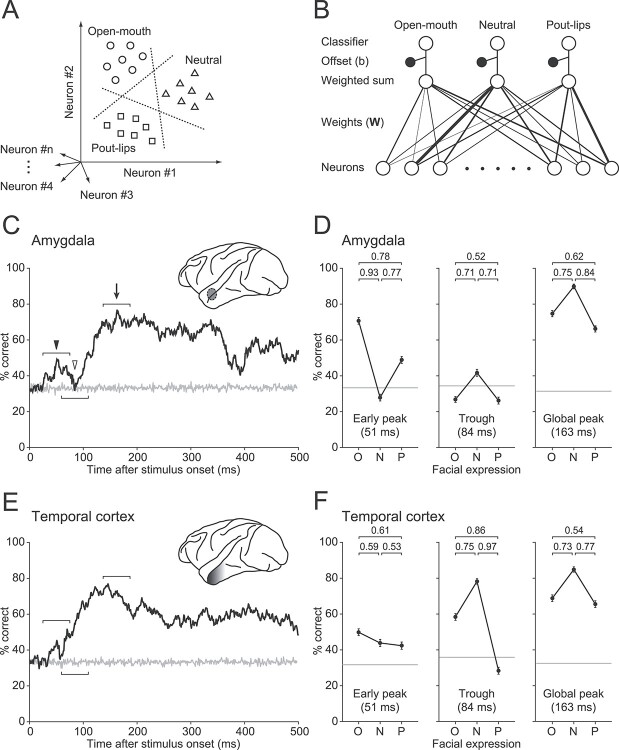
Population discrimination ability assessed by linear classification. A) Discrimination ability of facial expressions by population responses. Each symbol indicates the response of a population of neurons to a given image in a given trial, and different symbol types denote responses to different facial expressions. A linear hyperplane (dotted line) that effectively separates one response cluster from the others was determined for each facial expression by a SVM procedure (see Materials and methods). B) Implementation of classifiers based on the weighted sum of responses. The linear hyperplane in A is defined by weights on each axis (determining the hyperplane orientation) and an offset from the origin. The weights are zero, positive, or negative. The final decision is made by selecting the classifier with the largest output. C) Time course of discrimination performance for the amygdala population. The average performance across 100 simulations is plotted along the time axis. In a single simulation, some of the data (80%) were randomly selected in each neuron and used to determine the weights. The remaining data (20%) were used to test classification performance. The gray line represents chance performance estimated by shuffling the data. Representative time windows are indicated by the filled arrowhead (early peak), open arrowhead (trough), and arrow (global peak). D) Performance profiles of the amygdala population at the representative windows. The performance accuracy (mean ± SEM across 100 simulations) is plotted for the open-mouth (O), neutral (N), and pout-lips (P) faces. Gray lines indicate the chance level estimated by shuffling the data. The pairwise AUC values are shown at the top of the panels. *E*, *F*) Time course and profiles for discrimination performance in the temporal cortex population. Conventions are the same as in C and D.

We then examined the time course of the sensitivity to the facial expressions using a 50-ms sliding time window for each individual neuron. We selected the width of 50 ms based on a previous study (Sugase et al. 1999) that revealed the time course of selective responses to facial expressions and identities in the temporal cortex using a 50-ms sliding window with 8-ms increments. In our analysis, we moved the window at 1-ms increments to maintain a high temporal resolution, because we were not sure how quickly the neurons would change their responses. We tested the statistical significance of the sensitivity with the Friedman test (main factor: facial expression). Note that latencies below 25 ms in Figs. 3 and 4 were computed for windows spanning both prestimulus and poststimulus periods. This yielded a time course of the *P* value for the effect of facial expression. We counted the number of facial expression-discriminating neurons (criterion: *P* < 0.05) at each point in time. We estimated false positives at statistical criteria of *P* = 0.05 and *P* = 0.01 from the null distribution (see above). Because no systematic changes were present in the false-positive estimation along the time course, we merged and averaged the percentile values across time windows. Additionally, we performed the same Friedman test analysis with a more conservative criterion of *P* < 0.01. We used these 2 statistical criteria to examine whether a slight change in the statistical criteria affected the results.

We applied a linear classification approach (Hung et al. 2005; Rust and DiCarlo 2010) to the population activity of the amygdala and the temporal cortex to assess how well they discriminated the facial expressions. This approach constructs 3 classifiers, each of which is trained to discriminate one of the 3 facial expressions (open mouth, neutral, pout-lips) from the others (one-versus-rest). Each classifier has a set of weight parameters that determines the contribution of individual neurons. During training, the classifiers are optimized to discriminate the 3 facial expressions by adjusting the weights. After training, for a given image, each classifier is used to independently compute a weighted sum of responses of individual neurons. At the decision stage, the classifier with the largest weighted sum is chosen as the category prediction for the image. All neurons, even those that respond only slightly differently to the facial expressions, can contribute to discrimination performance with varying effectiveness. We used a sliding time window (50-ms width, 1-ms step) to examine the time course of the discrimination performance. For cross-validation purposes (see below), we needed a set of the responses for 10 trial repetitions. Among all face-responsive neurons, 100 amygdala neurons and 113 temporal cortex neurons met this criterion and were subjected to the analysis.

For each time window, we independently made different sets of classifiers. To implement them for a particular time window, we first counted the number of spikes in the time window for a given trial for each neuron and produced an array of spike counts for a population of neurons (response vector). For each stimulus, the response vector **x** can be plotted as a single point in a high-dimensional space, in which each axis represents the response strength of different neurons. The spike counts were separately normalized within each neuron (the maximum response was set to 1; e.g. actual spike counts of [0, 1, 2, 3, 4] were converted into a response vector of [0, 0.25, 0.5, 0.75, 1]) to compensate for differences in FRs across neurons. For other trials using the same stimulus, additional data points were plotted near the first one, but with some fluctuations. Likewise, other clusters might appear for other stimuli. Then, we searched for a linear hyperplane that separated the set of clusters corresponding to a particular facial expression from those corresponding to the other expressions (one-versus-rest classification). The computation of a linear classifier took the following form:

$$ f(x)={w}^Tx+b, $$

where ***x*** is a response vector, ***w*** is a weight vector that defines the hyperplane, and *b* is the offset of the hyperplane from the origin. We applied a support vector machine (SVM) procedure to determine the weight vector and the offset so that distances between the hyperplane and its nearest data points were maximized. We used the LIBSVM library (Chang and Lin 2011) to search for the optimal parameters (available at https://www.csie.ntu.edu.tw/∼cjlin/libsvm/). As library settings, we employed a linear kernel, the C-SVC algorithm, and a cost parameter of 0.125. We employed a portion of the neuronal data (8 out of 10 trials) for this training. Thus, a total of 72 samples (8 trials × 9 face images) of the response vector were used to determine the parameters. The remaining 18 samples (2 trials × 9 face images) were used to test classification performance (the 5-fold cross-validation, see below).

For each classifier (open mouth, neutral, and pout-lips), a weighted sum was separately computed for a response vector of a particular sample. After adding an offset, a positively or negatively weighted sum indicated that the data point was on one or the other side of the hyperplane in the high-dimensional response space. A large weighted sum was computed for data points that were far from the hyperplane (i.e. a large positive value corresponded to robustly correct classification). Thus, the weighted sums were compared and the classifier with the largest output was chosen as the prediction for the population. Performance was assessed by calculating the proportion of correct predictions across the 18 samples for cross-validation. To determine the temporal evolution of the performance, we repeated this procedure along the time axis by moving a 50-ms time window. Note that the performance does not reflect the effects of response covariation in the neurons (noise correlation) because the neuronal responses were not simultaneously recorded.

We equalized the number of neurons used for the classification when comparing the performance between the amygdala and the temporal cortex, because a greater number generally results in higher performance (Rust and DiCarlo 2010). In a single simulation, all 100 amygdala neurons tested with 10 trials were used, and 100 neurons were randomly selected from the 113 temporal cortex neurons tested with 10 trials. We repeated this simulation 100 times at each time point, and the mean correct rates across the simulations were plotted along the time axis. Chance levels of performance were estimated with null distributions generated by shuffling the stimulus–response relationships.

In the classification approach, we used receiver operating characteristic (ROC) analysis to quantify the degree to which the correct rate distributions differed across the facial expressions (Green and Swets 1966). ROC analysis produces a metric called the area under the curve (AUC) that represents the degree of separation between 2 different distributions (e.g. 0.5, totally overlapped; 1.0, perfectly separated). Note that AUC values smaller than 0.5 were converted to those greater than 0.5 by reflecting the values with respect to 0.5 (e.g. an AUC value of 0.45 was converted to 0.55).

### Test for interaction between SF and size tunings

For a subset of amygdala neurons (*n* = 35), we tested the effects of stimulus size on neuronal tuning for image-based SF by presenting a series of bandpass-filtered faces (center SF: 2.0, 2.8, 4.0, 5.7, 8.0, 11.3, 16.0 cycles/image) with different sizes (3.8° × 3.8°, 5.4° × 5.4°, 7.7° × 7.7°, 11.0° × 11.0°, 15.3° × 15.3°). Details of the analysis are described in our previous paper (Inagaki and Fujita 2011). In short, we exploited the difference in SF bandwidth between the subcortical and cortical pathways (Miller et al. 1980; Vuilleumier et al. 2003) and evaluated the relative contribution of the 2 pathways to the responses. The narrow bandwidth renders the subcortical pathway responses sensitive to retina-based SF (cycles/degree), while the broader bandwidth renders responses of the cortical pathway sensitive to image-based SF (cycles/image). For a filtered face that has a particular center image-based SF, the center retina-based SF depends on the stimulus size on the monitor. The larger the stimulus size, the lower the center retina-based SF, because dividing image-based SF (cycles/image) by stimulus size (degrees) produces retina-based SF (cycles/degree). By definition, the center image-based SF of the filtered face is independent of the stimulus size. This is our rationale for manipulating stimulus size to dissociate the 2 types of SFs. We calculated a shift index from SF tuning curves at different stimulus sizes to characterize how the preferred image-based SF changes across stimulus sizes (Inagaki and Fujita 2011). When the shift index is 1, the preferred image-based SF changes so as to be proportional to the stimulus size. This means that the preferred retina-based SF does not change across stimulus size (i.e. it is ideally tuned for retina-based SFs; see Fig. 6B). If the shift of the preferred image-based SF is larger than expected, based on the ideal retina-based SF tuning, the shift index becomes larger than 1. When the shift index is 0, the preferred image-based SF does not change across stimulus sizes (i.e. it is ideally tuned for image-based SFs). If the preferred image-based SF changes, but the direction of the shift is opposite to that based on the ideal retina-based SF tuning, the shift index becomes a negative value. We used a 500-ms fixed window for this analysis because a 50-ms sliding window was insufficient to gain reliable estimates of the effects.

### Histological verification of recording sites

We performed histological analysis in monkey SA to verify the recording sites in the amygdala and temporal cortex. After making microlesions using an electric current (10 μA, 10 s or 20 s, electrode negative) in these areas, the monkey was deeply anesthetized with an overdose of sodium pentobarbital (100 mg/kg, i.p.) and transcardially perfused with 0.1 M phosphate-buffered saline (PBS, pH 7.4) and 4% paraformaldehyde. The brain was immersed in a graded series of sucrose solutions (10%–30%), frozen, and cut into 80-μm coronal sections. The sections were stained for Nissl substance with cresyl violet. Recording sites were reconstructed using the position of the lesions (for photomicrographs, see Inagaki and Fujita 2011) and the readings of the electrode manipulator.

## Trans-synaptic tracing experiments

We explored the source of subcortical afferents to the amygdala by injecting rabies virus as a retrograde trans-synaptic tracer. We were particularly interested in obtaining evidence for a multisynaptic route, connecting the amygdala with the pulvinar and superior colliculus, which was previously implicated in the subcortical face processing pathway (Morris et al. 2001; Johnson 2005; Pegna et al. 2005; Tamietto and de Gelder 2010). Rabies virus is taken up by axon terminals, but not by passing fibers, and retrogradely transported to the cell bodies (first-order labeling). Then, the virus is replicated, released from the cell bodies or dendrites, taken up by axon terminals of presynaptic cells, and transported to their cell bodies (second-order labeling). With a longer survival period, the number of synapses across which the virus is transported increases: uptake and transport times are pathway specific. We used a survival period of 2 days, which was thought to allow for only first- and second-order labeling (Kelly and Strick 2000; Ugolini 2011; see also Results).

### Surgery for tracer experiments

The monkeys (HE, SE) were initially sedated with ketamine hydrochloride (5 mg/kg, i.m.) and xylazine hydrochloride (0.5 mg/kg, i.m.) and then anesthetized with ketamine hydrochloride and propofol (10–15 mg/kg/h and 5–7.5 mg/kg/h, respectively, i.v.). An antibiotic (ceftazidime, 25 mg/kg, i.v.) and an analgesic (meloxicam, 0.2 mg/kg, s.c.) were administered at the start of anesthesia. Lactated Ringer’s solution was infused (i.v.) during surgery. After craniotomy over the right parietal cortex, a 10-μL Hamilton microsyringe was inserted toward the lateral part of the amygdala with the aid of a magnetic resonance imaging (MRI)-guided navigation system (Brainsight Primate; Rogue Research). Postoperatively, the monkeys were treated with an antibiotic (cefotiam hydrochloride, 20 mg/kg) for 2 days.

### Viral injection

The rabies virus (CVS-26 strain, 3.0 × 10^8^ focus-forming units/mL) was obtained from the Centers for Disease Control and Prevention (Atlanta, GA) and was donated by Dr Satoshi Inoue (The National Institute of Infectious Diseases, Tokyo, Japan). Injection tracks were made in the lateral part of the amygdala: 3 in monkey HE and 2 in monkey SE. Along each injection track, we injected a small volume of viral solution (0.5 μL) at 2 different depths in monkey HE and at a single depth in monkey SE. After completing the injections, the bone flap was repositioned and cemented with dental resin, and the scalp incision was sutured.

### Histology for tracer experiments

The monkeys were subsequently kept alive for approximately 2 days (52 h for monkey HE, 50 h for monkey SE). Then, they were deeply anesthetized with an overdose of sodium pentobarbital (50 mg/kg, i.v.) and transcardially perfused with 0.1 M PBS followed by 10% formalin in 0.1 M phosphate buffer (PB). The brains were removed and postfixed in the same fresh fixative overnight at 4°C and saturated with 30% sucrose in 0.1 M PB (pH 7.4). Coronal sections were then cut serially at 50-μm thickness on a freezing microtome. Every 10th section was processed for immunohistochemical staining for the rabies virus by means of the standard avidin–biotin–peroxidase complex method (Ishida et al. 2018). Following immersion in 1% skimmed milk, the sections were incubated overnight with a rabbit antibody against rabies virus (donated by Dr Satoshi Inoue) in 0.1 M PBS containing 0.1% Triton X-100 and 1% normal goat serum. The sections were then placed in the same fresh incubation medium containing biotinylated goat anti-rabbit IgG antibody (diluted at 1:200; Vector Laboratories), followed by the avidin–biotin–peroxidase complex kit (ABC Elite, Vector Laboratories). To visualize the reaction product, the sections were reacted in 0.05 M Tris-HCl buffer (pH 7.6) containing 0.04% diaminobenzidine, 0.04% nickel chloride, and 0.002% hydrogen peroxide. These sections were counterstained with 0.5% neutral red, mounted onto gelatin-coated glass slides, dehydrated, and coverslipped. Additionally, adjacent sections were mounted onto gelatin-coated glass slides and Nissl-stained with 1% cresyl violet.

### Data analysis for tracer experiments

Our analysis focused on determining whether the superior colliculus demonstrated second-order labeling, and if so, in what layer(s). According to May (2006), the superior colliculus is divided into the stratum zonale/stratum griseum superficiale (SZ/SGS), stratum opticum (SO), stratum griseum intermedium/stratum album intermedium, stratum griseum profundum, and stratum album profundum (SAP). Rabies-labeled cell bodies in the superior colliculus were plotted using a Neurolucida system (MBF Bioscience) coupled to a microscope (Eclipse 80i; Nikon) with a motorized stage (Ludl Electric Products) and a charge-coupled device camera (CX9000; MBF Biosciences). Sections of the thalamus and superior colliculus were photographed using a microscope (NanoZoomer S60; Hamamatsu Photonics or Eclipse E800M; Nikon). The micrographs were adjusted for brightness and contrast using Photoshop Elements 9 (Adobe). These manipulations were applied to the entire image. Nissl-stained sections were used to outline the brain regions, nuclei, and layers. We adopted the atlas by Olszewski (1952) to identify thalamic nuclei and subnuclei.

## Results

By conducting 2 separate types of experiments, we tested the dual-route hypothesis that amygdala neurons receive rapid signals regarding facial expressions via a subcortical pathway. In physiological experiments, we examined whether amygdala neurons differentially responded to facial expressions with a short latency. We analyzed the responses of face-responsive amygdala neurons to 9 face images in which 3 monkeys provide 3 different expressions, i.e. open mouth, neutral, and pout-lips (Fig. 1A). For comparison, we examined the responses of visual temporal cortex neurons of the same animals to the same set of stimuli. In anatomical experiments, we tested another prediction of the dual-route hypothesis, namely that the retinorecipient “visual” layers of the superior colliculus connect to the amygdala with a few synaptic relays. We injected rabies virus, which permits retrograde trans-synaptic transport, into the amygdala. We examined the distribution of labeled neurons at 2 days post-injection when at a minimum, first-order (monosynaptic) and second-order (disynaptic) transport would have occurred.

### Database of single-unit recordings

We obtained recordings from 104 face-responsive neurons in the amygdala (77 from monkey SA, 27 from monkey KU; see Materials and methods for definition of face-responsive neurons). Recordings in the amygdala covered the entire mediolateral extent and the anteroposterior extent from A20 to A25. Histological examination in one of the monkeys (monkey SA) revealed that the face-responsive neurons were primarily found in the lateral nucleus, with a few examples observed in adjacent parts of the central nucleus and the lateral part of the basal nucleus (Fig. 1B). The neurons were distributed throughout the dorsal two-thirds of the lateral nucleus. We also analyzed 116 face-responsive neurons in the temporal visual cortex (68 from monkey SA, 48 from monkey KU). These were located mainly in the fundus and lower bank of the superior temporal sulcus and partly in the inferior temporal gyrus. The recorded regions corresponded to cytoarchitectonic areas TE1, TE2, TEa, IPa, and PGa of Baylis et al. (1987).

### Short-latency, expression-discriminating responses in single amygdala neurons

A significant minority of the amygdala neurons demonstrated short-latency, differential responses to the 3 facial expressions. The early responses around 55 ms after stimulus onset in the 2 neurons shown in Fig. 2 exhibited statistically significant differences in FRs across different facial expressions (Fig. 2B: Friedman test, *n* = 10 trials, df = 2, χ^2^ = 9.30, *P* = 0.0096; Fig. 2D: Friedman test, *n* = 10 trials, df = 2, χ^2^ = 8.16, *P* = 0.017). The neuron in Fig. 2B responded best to open-mouth faces (inset; the orange line, corresponding to open-mouth faces, is higher than the lines corresponding to the other expressions), while the neuron illustrated in Fig. 2D initially responded least to open-mouth faces (inset; the orange line is lower than the others). Note that in this analysis, we focused exclusively on a 50-ms time window centered at 55 ms after stimulus onset (the “early window”; 30–80 ms) because the fastest reported response of monkey pulvinar neurons to faces and face-like patterns is 30 ms (Nguyen et al. 2013), and early amygdala responses would necessarily occur at a slightly greater latency. Fig. 2A and C shows peristimulus time histograms and raster plots of these neurons over the entire period of stimulus presentation.

Across the 104 amygdala neurons tested, the number of neurons with differential responses to the facial expressions (Friedman test, tested with *n* ≥ 6 trials, *P* < 0.05) increased slightly around 55 ms after stimulus onset (Fig. 3A). To evaluate the statistical significance of this brief, small increase, we shuffled the data to create a null distribution (*n* = 1,000 simulations) and then determined its 95th and 99th percentiles. The number of expression-differentiating neurons was judged to be significant at a 0.05 or 0.01 level if it was greater than the values at these percentiles (0.05, dashed line; 0.01, dotted line). In the early window centered at 55 ms (arrowhead), the number of expression-differentiating neurons was 10, which surpassed the significance level of 0.05, indicating that a small but significant number of amygdala neurons discriminated the facial expressions during this time period. The single-neuron responses were typically not specific for only one expression but were elicited by multiple expressions with different strengths. Around the 55-ms time point, the number of expression-differentiating neurons reached a significance level of 0.05 in several windows. The number dropped to a prestimulus level for a few tens of milliseconds, then increased substantially beginning 100 ms after stimulus onset and lasted for nearly the entire stimulus presentation. Similar results were obtained for a smaller subset of neurons that were judged to be expression-differentiating with a more stringent criterion (Friedman test, 104 neurons tested with *n* ≥ 6 trials, *P* < 0.01; Fig. 3B). The results revealed that the amygdala transmitted a fast and transient signal related to facial expression, with a latency of around 50 ms.

By contrast, for the 116 temporal cortex neurons recorded from the same animals (Fig. 3C and D), the number of expression-discriminating neurons surpassed significant levels around 70 ms after stimulus onset, slightly later than the initial increase in the amygdala population (compare Fig. 3A and B and Fig. 3C and D). Signals from the temporal cortex are unlikely to contribute to the short-latency response observed in the amygdala. They may contribute to a later component of expression-discriminating responses in the amygdala because the increase in the number of expression-discriminating neurons in the temporal cortex preceded the second, larger buildup of the amygdala population.

### Discrimination performance of ensembles of amygdala neurons

Although a significant minority of the amygdala neurons differentially responded to the 3 facial expressions around 55 ms after stimulus onset, it is unclear how robustly amygdala neurons as a population encode facial expressions with such short latency. To investigate this issue, we applied a linear classification approach to population activity (Hung et al. 2005; Rust and DiCarlo 2010), which allowed us to evaluate information about the facial expressions that were encoded by ensembles of amygdala neurons (see Materials and methods). This approach assessed how linear hyperplanes separated the different stimulus categories (i.e. 3 facial expressions) within a high-dimensional space spanned by the response strength of each neuron (Fig. 4A). We constructed 3 classifiers, each for a different facial expression (Fig. 4B). Each classifier collected the responses of neurons with different connection weights and produced a variable (the weighted sum of responses) that represented the likelihood of an assigned facial expression. We analyzed the discrimination performance of the classifiers as a function of time, using a 50-ms sliding window with 1-ms steps.

Linear classifiers constructed for the amygdala were able to read out information about the open-mouth faces in a window around 50 ms after stimulus onset. The time course of the overall performance averaged across the 3 classifiers (Fig. 4C) revealed a small early peak (filled arrowhead; window center, 51 ms) and a later, global peak (arrow, 163 ms). The delay between the 2 peaks (112 ms) was longer than the width of the sliding window (50 ms), indicating that the temporal resolution of this analysis was sufficient. The 2 peaks were separated by a trough (open arrowhead, 84 ms) during which performance dropped to chance level. At the early peak, performance was highest for the open-mouth classifier that discriminated open-mouth faces from the other faces, indicating rapid detection of open-mouth faces by a population of amygdala neurons (Fig. 4D, left panel; open mouth vs. neutral: AUC = 0.93, open mouth vs. pout-lips: AUC = 0.78). At the trough and global peak, performance was higher for the neutral classifier than for the others (Fig. 4D, middle and right panels; neutral vs. open mouth: AUC = 0.71 for trough, AUC = 0.75 for global peak; neutral vs. pout-lips: AUC = 0.71 for trough, AUC = 0.84 for global peak). Higher performance for the open-mouth faces was thus prominent only at the early peak.

### Comparison of performance profiles between the amygdala and temporal cortex

By contrast, linear classifiers for neurons in the anterior temporal visual cortex of the same animals did not exhibit better performance for open-mouth faces (Fig. 4E and F). In the early-peak window (Fig. 4F, left panel), performance with the open-mouth classifier was comparable to that of both the neutral classifier (open mouth vs. neutral: AUC = 0.59) and the pout-lips classifier (open mouth vs. pout-lips: AUC = 0.61). Because visual cortical projections to the amygdala originate mainly from the anterior part of the temporal cortex (Aggleton et al. 1980), these results are consistent with the idea that rapid detection of open-mouth faces in the amygdala is mediated by signals from a pathway that bypasses the visual cortex (LeDoux 2000; Morris et al. 2001; Pegna et al. 2005; Vuilleumier 2005).

At the global peak, the neutral classifier performed better than the emotional (i.e. open mouth and pout-lips) classifiers both in the amygdala (Fig. 4D, right panel) and temporal cortex (Fig. 4F, right panel). Similar profiles in the later period suggest that the amygdala and the temporal cortex may share the outputs of the ventral cortical pathway.

### Excitatory and suppressive components in rapid responses of amygdala neurons

**Figure 5:**
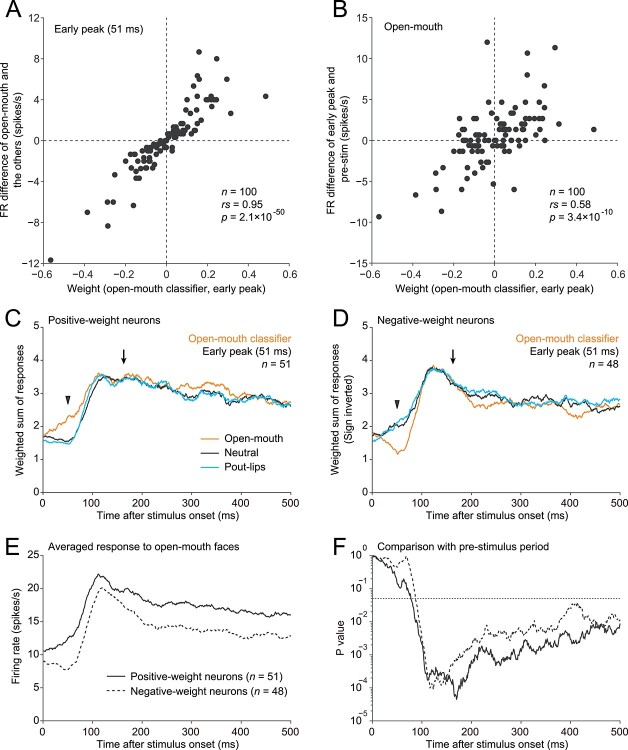
Early-peak weight of the open-mouth classifier constructed for the amygdala population. A) Relationship between weight and the preference for open-mouth faces. Differences in mean FRs for open-mouth faces and the other faces were calculated at the early-peak window (centered at 51 ms) and are plotted along the *y*-axis. B) Relationship between the weight and strength of the early response to open-mouth faces. Differences in mean FRs for open-mouth faces between the early-peak and pre-stimulus (−50 to 0 ms) windows are plotted along the *y*-axis. C, D) Time course for the weighted sum of responses (50-ms sliding window) across amygdala neurons with positive (C) and negative weights (D) (open mouth, orange; neutral, black; pout-lips, cyan). One neuron had a weight of zero and was excluded from the analysis. The weighted sums were averaged across 100 simulations. Standard errors of the mean are shaded but are too small to visualize. E) Time course of the averaged responses to open-mouth faces across amygdala neurons with positive (solid line) and negative weights (dashed line). F) Time course of statistics in comparison with the pre-stimulus level. For each group (positive weight, solid line; negative weight, dashed line) at a given time window, the distribution of the FRs across neurons was compared with that in the prestimulus window (−50 to 0 ms) (Wilcoxon signed-rank test). The *P* value curves initially dropped below 0.05 (dotted horizontal line), at 78 ms for the positive-weight group and at 87 ms for the negative-weight group.

We sought to identify the mechanisms involved in rapid threat detection by analyzing the early-peak weight of the open-mouth classifier constructed for the amygdala population (Fig. 5). Each neuron in a classifier had a single weight with either a positive or negative value. As expected from the SVM procedure, neurons with a positive weight contributed to the discrimination by responding more strongly to the open-mouth faces than to the other faces, while neurons with a negative weight exhibited weaker responses to the open-mouth faces than to the other faces (Fig. 5A; Spearman’s rank correlation, *n* = 100, rs = 0.95, *P* = 2.1 × 10^−50^). Moreover, compared with prestimulus levels (FRs in the −50 to 0 ms window), positive- and negative-weight neurons showed excitatory and suppressive responses, respectively, as a result of increasing and decreasing their FRs after the presentation of open-mouth faces (Fig. 5B; Spearman’s rank correlation, *n* = 100, rs = 0.58, *P* = 3.4 × 10^−10^). The strong correlation shown in Fig. 5A was self-evident from the nature of the analysis, but the correlation between the weight and the excitation/suppression shown in Fig. 5B was not self-evident, i.e. not automatically deduced from the data shown in Fig. 5*A*. The large absolute values of the weight appeared in both excited and suppressed neurons, indicating that both excitation and suppression in response to the open-mouth faces in the amygdala contributed to early discrimination relative to the other faces.

Given these qualitative differences, we divided the amygdala neurons into positive-weight (*n* = 51) and negative-weight (*n* = 48) groups based on the weight of the open-mouth classifier at the early peak. Note that one neuron had a weight of zero and was excluded from the analysis. We then separately plotted the time course of the open-mouth classifier’s weighted sums to evaluate how well this classifier discriminated the open-mouth faces from the other faces as a function of time after stimulus onset. At the early peak, as expected, the weighted sum was stronger in response to the open-mouth faces than to the other faces in the positive-weight group (Fig. 5C, arrowhead) and weaker in the negative-weight group (Fig. 5D, arrowhead). At the later global peak, however, preference for the open-mouth faces in the outputs disappeared in both positive-weight (Fig. 5C, arrow) and negative-weight groups (Fig. 5D, arrow). Thus, the mechanisms underlying the rapid threat detection in the amygdala function only transiently and lose their effect in later time periods.

We plotted the time course of the averaged response across neurons separately for the positive- and negative-weight groups using the 50-ms sliding time window (Fig. 5E). We compared the response distribution in each time window with that in the prestimulus period (−50 to 0 ms) (Wilcoxon signed-rank test) and obtained the time course of the *P* value (Fig. 5F). Response latency (here defined as the time point when the *P* value first dropped below 0.05) was shorter in the positive-weight group (78 ms) than in the negative-weight group (87 ms), indicating that the positive-weight neurons were driven faster than the negative-weight neurons.

**Figure 6:**
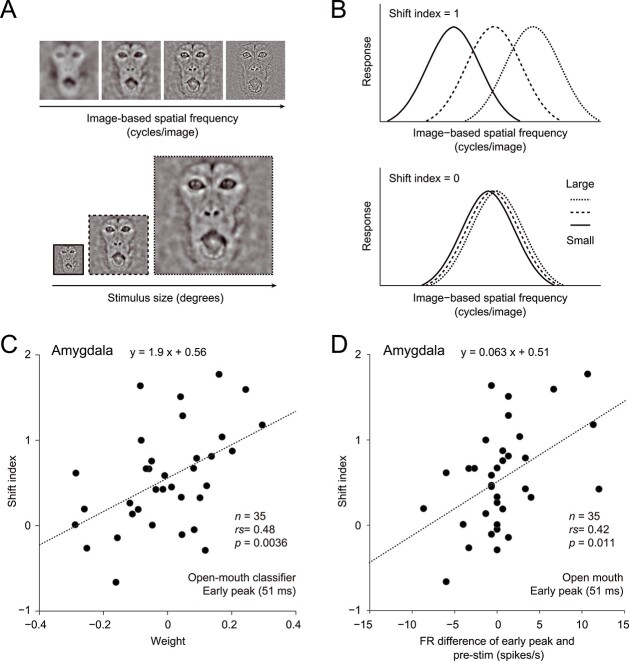
Spatial reference frames of face-responsive neurons in the amygdala. A) Visual stimuli for testing the effects of stimulus size on SF tuning. A stimulus set consisted of 35 images (all combinations of 7 SFs and 5 sizes; for details, see Materials and methods) in actual experiments. B) SF tuning curves of neurons ideally tuned for retina-based SF (cycles/degree) (upper panel, shift index = 1) and those for image-based SF (cycles/image) (lower panel, shift index = 0). The shift index was calculated from the effects of stimulus size on SF tuning and thus represents the SF tuning type (for details, see Materials and methods). C) Relationship between weight and shift index in the amygdala. Mean values across 100 simulations are plotted for the weight of the open-mouth classifier at the early peak. D) Relationship between the strength of the early response to open-mouth faces and the shift index in the amygdala. Differences in mean FRs for open-mouth faces between the early-peak and prestimulus (−50 to 0 ms) windows are plotted. The dotted lines in (C) and (D) represent regression lines.

We next probed the contribution of the subcortical and cortical pathways to the open-mouth face detection by analyzing neuronal SF selectivity. We previously examined reference frames for SF in face-responsive neurons by testing the effects of stimulus size on SF selectivity (Fig. 6A) (Inagaki and Fujita 2011). Amygdala neurons show a variety of shifts in their preferred image-based SF (cycles/image) depending on stimulus size. In a subpopulation of amygdala neurons, the preferred image-based SF changes proportionally with the stimulus size (Fig. 6B, upper panel), so that the preferred retina-based SF (cycles/degree) is kept constant across stimulus sizes. In another population, the preferred image-based SF is kept constant across stimulus sizes (Fig. 6B, lower panel), a common property of temporal cortex neurons (Inagaki and Fujita 2011). We quantified the degree of SF tuning shifts to construct a shift index for the purpose of characterizing neurons’ SF tuning types (shift index = 1, ideally tuned for retina-based SF; shift index = 0, ideally tuned for image-based SF; see Materials and methods). We computed the shift index using a fixed 500-ms window after the stimulus onset. We also tried this analysis using the 50-ms sliding time window but failed to obtain reliable results because the parameter fitting of this analysis did not converge to robust estimates with this small window.

For the amygdala open-mouth classifier, weight at the early peak was correlated with the SF tuning type across neurons (retina-based vs. image-based, characterized by the shift index); positive-weight neurons had retina-based SF tuning, while negative-weight neurons had image-based SF tuning (Fig. 6C; Spearman’s rank correlation, *n* = 35, rs = 0.48, *P* = 0.0036; regression line, *y* = 1.9 *x* + 0.56). Additionally, the response type (excitation vs. suppression) was correlated with the SF tuning type (Fig. 6D; Spearman’s rank correlation, *n* = 35, rs = 0.42, *P* = 0.011; regression line, *y* = 0.063 *x* + 0.51). The excited neurons were tuned for retina-based SF, indicating that early excitation is likely mediated by subcortical processing. The suppressed neurons had image-based SF tuning, suggesting that they receive slow inputs from the temporal visual cortex. Note that the shift index reflected averaged inputs to the neuron during visual stimulation, most likely including both subcortical and cortical inputs. Therefore, suppression can accompany cortex-like properties if later sustained excitatory responses of early suppressed neurons are dependent on cortical processing. Based on these results, we developed a hypothetical model of the amygdala in which early excitation and suppression play different roles in open-mouth face detection, as mentioned below (see Discussion). No correlation between weight and SF tuning type was observed in the temporal cortex population (51 ms, open-mouth classifier, *n* = 37, rs = 0.037, *P* = 0.83) or at the global peak within the amygdala population (163 ms, open-mouth classifier, *n* = 35, rs = 0.24, *P* = 0.16). The subcortical pathway may shape early excitation by influencing a population of amygdala neurons, while the cortical pathway may contribute to another amygdala population that is initially suppressed for a short while.

### Retrograde labeling in the pulvinar and superior colliculus

**Figure 7:**
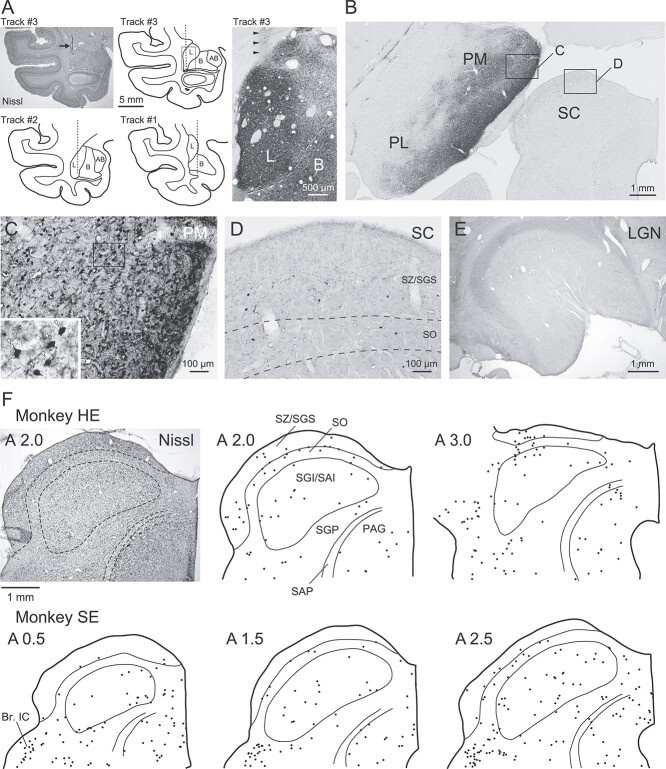
Retrograde neuron labeling in the pulvinar and superior colliculus following rabies injection into the amygdala. A) Injection tracks (left and middle panels) and sites (right panel) in the amygdala. The arrow in the Nissl-stained section indicates the injection needle track. The dotted lines represent the injection tracks. The region in the right panel corresponds to the solid rectangle in the coronal section of track #3, and arrowheads show the injection track. L, lateral nucleus of the amygdala; B, basal nucleus of the amygdala; AB, accessory basal nucleus of the amygdala. B) Low-power photomicrograph of retrograde labeling in the pulvinar and superior colliculus. PM, medial pulvinar; PL, lateral pulvinar; SC, superior colliculus. C, D) Higher-power photomicrographs of the pulvinar (C) and superior colliculus (D). The regions in (C) and (D) correspond to solid rectangles marked as “C” and “D,” respectively, in (B). Inset shows labeled neurons (indicated by arrowheads) at higher magnification. SZ, stratum zonale; SGS, stratum griseum superficiale; SO, stratum opticum. E) Low-power photomicrograph of the LGN. F) Plots of retrogradely labeled cell bodies in the superior colliculus following rabies injections into the amygdala in 2 monkeys (HE and SE). The rostrocaudal level of each plot is indicated on the upper left. Nissl-stained sections were used to outline the layers (upper left panel). SZ, stratum zonale; SGS, stratum griseum superficiale; SO, stratum opticum; SGI, stratum griseum intermediale; SAI, stratum album intermediale; SGP, stratum griseum profundum; SAP, stratum album profundum; PAG, periaqueductal gray; Br. IC, brachium of the inferior colliculus.

We examined monosynaptic and multisynaptic connections that link the amygdala with the pulvinar and superior colliculus and are implicated in the subcortical pathway for emotional face processing (Morris et al. 2001; Johnson 2005; Pegna et al. 2005; Tamietto and de Gelder 2010). In 2 monkeys (monkeys HE and SE), we injected rabies virus as a retrograde trans-synaptic tracer into the lateral part of the amygdala (Fig. 7A), which is the entry point for a variety of sensory afferents from subcortical and cortical areas (Jones and Burton 1976; Aggleton et al. 1980; Norita and Kawamura 1980; Amaral et al. 1992; Cheng et al. 1997; Romanski et al. 1997; Stefanacci and Amaral 2000). The injection tracks indicated that the tip of the microsyringe was positioned mainly at the lateral amygdala (Fig. 7A, left and middle panels). While neurons in the lateral amygdala were strongly and extensively labeled at the injection site below the track trace (indicated by arrowheads in Fig. 7A, right panel), it was difficult to delineate the exact extent of the virus injection separately from the local neurons infected by the virus. This was because we injected a very small amount of the virus and detected the virus proteins replicated in the neurons, not the injected virus itself.

Strong retrograde labeling was observed in the medial part of the pulvinar (Fig. 7B), where both cell bodies and fibers were densely labeled (Fig. 7C). The survival period of 2 days used in this study (52 h for monkey HE and 50 h for monkey SE) was thought to allow the rabies virus to infect neurons with no more than 2 synaptic relays from the injection site (Kelly and Strick 2000; Ugolini 2011). We verified whether or not rabies infection across 3 synapses occurred in our materials by testing the distribution of retrograde labeling in visual area V4. The lateral part of the amygdala receives projection fibers from the anterior aspect of the temporal cortex (Stefanacci and Amaral 2000), to which V4 neurons in the supragranular layers send projection fibers (Desimone et al. 1980). The labeled neurons in V4 were seen in the supragranular layers, but not in the granular or infragranular layers, indicating that virtually no trisynaptic infection occurred. Thus, the neuronal labeling in the pulvinar likely resulted from either monosynaptic or disynaptic transport from the amygdala.

A significant number of neurons were also labeled in the superior colliculus of both monkeys, though they were much sparser than the labeled neurons in the pulvinar (Fig. 7D). Because previous studies using non-trans-synaptic tracers revealed no direct projection from the superior colliculus to the amygdala, we assume that the labeling in the superior colliculus resulted from disynaptic transport via relay areas including the pulvinar. These labeled neurons were distributed across all layers throughout the rostrocaudal extent of the superior colliculus (Fig. 7F). Of particular note was that the labeled layers included the retinorecipient superficial layers, namely the SGS and SO. We found no, or very few, retrogradely labeled neurons in the LGN (only 2 across 10 sections in monkey HE and none in monkey SE), suggesting very weak, if any, direct or disynaptic projections from the LGN to the lateral amygdala (Fig. 7E). We found no clear differences between the 2 monkeys in the results described above, despite the fact that the protocol details differed slightly (i.e. the number of injection tracks, the number of viral deposits, and the survival period after viral injection; see Materials and methods). The results provide evidence for disynaptic connections from the superior colliculus via another area (presumably including the pulvinar) to the amygdala (see Discussion).

**Figure 8:**
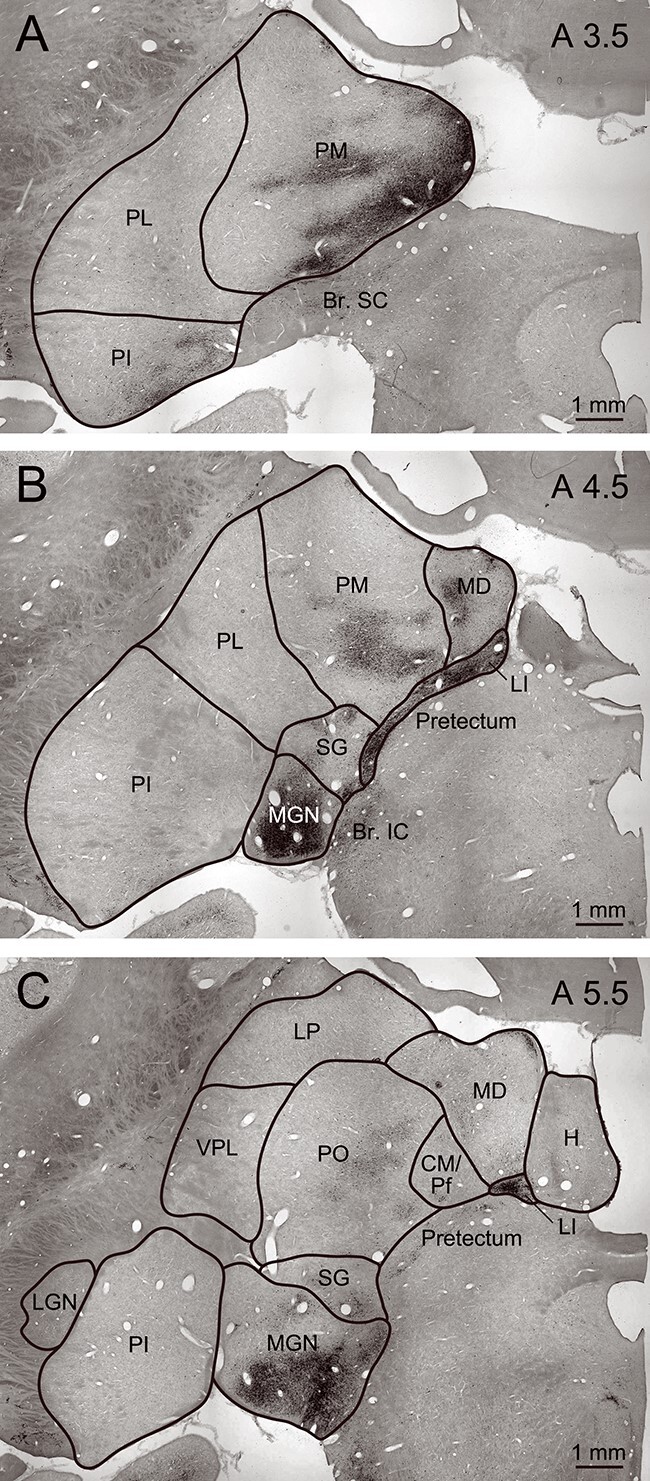
Retrograde labeling in thalamic nuclei. Low-power photomicrographs were taken at A3.5 (A), A4.5 (B), and A5.5 (C). Nissl-stained sections were used to outline the borders. PM, medial pulvinar; PL, lateral pulvinar; PI, inferior pulvinar; Br. SC, brachium of the superior colliculus; MD, mediodorsal nucleus; LI, nucleus limitans; SG, suprageniculate nucleus; MGN, medial geniculate nucleus; Br. IC, brachium of the inferior colliculus; H, habenula; CM/Pf, centre médian/parafascicular complex; PO, oral pulvinar; LP, lateral posterior nucleus; VPL, ventroposteriolateral nucleus; LGN, lateral geniculate nucleus.

The labeled neurons in the pulvinar were widely distributed across the medial, inferior, and lateral subdivisions and were most prominent in its ventromedial portion (Figs. 7B and 8). At more rostral levels, dense and massive labeling was observed in the medial geniculate nucleus and nucleus limitans (Fig. 8B and C). Labeled neurons were also found in other thalamic nuclei, including the oral pulvinar, mediodorsal nucleus, suprageniculate nucleus, and centre médian/parafascicular complex, and in the habenula (Fig. 8B and C). These distributions were essentially consistent with observations in previous studies in which conventional retrograde tracers were injected into the amygdala (Norita and Kawamura 1980; Elorette et al. 2018). In addition to these labeled neurons in the thalamus, we found sparsely labeled neurons in the pretectal region, including the brachium of the superior colliculus (Fig. 8A). Labeling was also observed in the nucleus of the brachium of the inferior colliculus (Figs. 7F and 8B).

## Discussion

### Rapid responses of amygdala neurons discriminate threatening faces

Here, based on single-unit recordings, we report for the first time the presence of short-latency, facial expression-sensitive responses in the amygdala. These single-neuron responses were typically not specific for only one expression in the early time window but were elicited by multiple expressions with different strengths. The absence of clearly specific responses might be the reason why previous studies (Kuraoka and Nakamura 2006; Gothard et al. 2007; Mormann et al. 2008; Rutishauser et al. 2011) failed to find early differential responses to facial expressions in the amygdala. The number of these neurons was small but statistically significant during the early window (30–80 ms after stimulus onset) when visual inputs from the pulvinar presumably first reach the amygdala (Nguyen et al. 2013).

The amygdala neurons as a population showed a robust ability to discriminate open-mouth faces from the other faces during the early window (Fig. 4D, left panel). A recent study reported that intracranial LFPs recorded in the human amygdala showed rapid and transient responses discriminating fearful faces from neutral and happy faces (Méndez-Bértolo et al. 2016), just as we showed here for single-neuron spiking activity. LFPs and spiking activity are dominated by different mechanisms; LFPs reflect synaptic input to a large number of neurons around a recording site, and spikes reflect outputs of the recorded neurons. Our finding that the population readout of single-unit responses showed the ability to differentiate open-mouth faces in an early time window suggests that the primate amygdala not only receives, but also relays, rapid signals of open-mouth faces decodable by downstream areas. As the open-mouth face is suggested to be a threatening expression in macaques (Hinde and Rowell 1962; Van Hooff 1962, 1967), this result suggests that brain areas downstream of the amygdala, such as the hypothalamus and midbrain periaqueductal gray, may rapidly read out information about threatening faces, thus triggering fast autonomic or hormonal responses or defensive behaviors (Amaral et al. 1992; LeDoux 2000).

In contrast to the early peak, the amygdala population response at the later global peak demonstrated improved discriminability for all of the classifiers (Fig. 4D, right panel). The performance was highest for the classifier that discriminated the neutral faces from the other faces. In this late period, the amygdala neurons differentiated neutral faces from emotional faces, regardless of whether they were threatening (open mouth) or affiliative (pout-lips). Although threatening and affiliative faces are associated with negative (threat-related) and positive (reward-related) signals, respectively, both likely increase the arousal level more strongly than neutral faces. The differentiation of emotional faces, either threatening or affiliative, from neutral faces in a later period might be useful to properly control the arousal level. Consistent with this idea is the finding that a subset of amygdala neurons showed similar sensitivity to both negative and positive signals (Belova et al. 2007). The amygdala may enhance the cortical visual processing based on both negative and positive affective signals via feedback connections (Vuilleumier 2015).

In the temporal cortex, the global peak window performance was highest for the neutral classifier (Fig. 4F, right panel), as was the case in the amygdala (Fig. 4D, right panel), suggesting that the same mechanism underlies later face representations in the temporal cortex and amygdala. Moreover, the significant performance of the neutral classifier was already apparent in the trough window (Fig. 4F, middle panel) for the temporal cortex population but not for the amygdala population (Fig. 4D, middle panel). The temporal order of the preference for the neutral classifier indicates that cortical processing is likely to contribute to the later representations in the amygdala, which is presumably useful for arousal computation.

While these results are consistent with the idea of a dual-route system in which rapid subcortical processing and slow cortical processing converge in the amygdala, a few uncertainties remain. Expression-discriminating responses are found in specific subregions of the temporal cortex (Hadj-Bouziane et al. 2008; Zhu et al. 2013; Taubert et al. 2020). Here we aimed to probe the major cortical contribution to the amygdala responses and recorded neurons only in the anterior part of the temporal cortex from which extensive visual cortical projections to the amygdala originate (Aggleton et al. 1980). We did not assess contributions by other facial expression-discriminating neurons outside of our sampling area, which would require us to identify face patches with functional MRI. An alternative approach to this issue will be to establish the contribution of the pulvinar to the early amygdala response by blocking neural activity in the pulvinar with pharmacological or chemical genetics techniques. Another question is the effects of stimulus type on the expression-discriminating responses of the amygdala and temporal cortex. Dynamic faces, rather than the static ones used in the current study, may reveal additional insight into the nature of expression-discriminating responses, given that these stimuli are often more effective at eliciting responses in the amygdala (Kuraoka and Nakamura 2006) and temporal cortex (Furl et al. 2012; Polosecki et al. 2013; Fisher and Freiwald 2015; Shepherd and Freiwald 2018). It remains to be determined whether temporal cortex neurons respond to dynamic faces as fast as to amygdala neurons.

### The dual-role hypothesis of subcortical processing

By testing the effect of stimulus size on SF selectivity, we revealed that the subcortical and cortical pathways play specific roles in rapid threat detection. In the amygdala, neurons that demonstrated early excitation or suppression showed retina- or image-based SF tuning, respectively (Fig. 6D). Early excitation is likely mediated by subcortical processing because of its link with retina-based SF tuning. Supporting this is the dissimilarity in performance profiles between the amygdala and temporal cortex at the early peak (Fig. 4D and F, left panels). Thus, even if fast signals from the temporal cortex exist, they likely provide only a minor contribution to early discriminatory signals in the amygdala.

**Figure 9:**
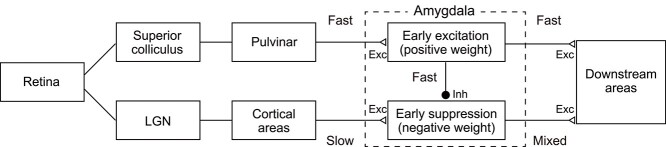
The dual-role model for the processing of threatening faces. Early excitation through the subcortical (colliculo–pulvino–amygdalar) pathway in a group of amygdala neurons mediates the rapid signaling of threats. Early suppression in another group of neurons originates from the subcortical pathway, is mediated by local inhibition, and enhances late-arriving excitatory inputs from the geniculo–cortical pathway via temporal contrast.

Assuming local inhibition within the amygdala (Ehrlich et al. 2009; Janak and Tye 2015), fast subcortical excitation could initiate early suppression in another group of amygdala neurons. If so, the correlation of early suppression and the temporal cortex-like property (i.e. image-based SF tuning) that we found suggests a convergence of subcortical and cortical processing in single amygdala neurons with a time delay (Fig. 9). Slower, sustained excitatory responses (most likely corresponding to cortical inputs) to the open-mouth faces rebound from the suppression and show a sharp rise in their response time course (Fig. 5D, orange line). We speculate that this potentially improves detection of facial information by downstream areas due to enhanced temporal contrast (Lee et al. 2013). Further experiments focusing on local inhibition within the amygdala may clarify this hypothetical dual role of the subcortical pathway in which both rapid signaling itself and its interaction with slower cortical signaling play roles in face processing. The former might be suitable to quickly begin preparations for behavioral reactions, whereas the latter might be used to confidently decide whether to trigger or quell these reactions.

### The colliculo–pulvino–amygdalar pathway and its relation to threat-signal processing

Here we presented the first direct evidence for neuronal projections from the superior colliculus through the pulvinar to the amygdala. Injections of rabies virus into the lateral part of the amygdala resulted in strong labeling in the pulvinar, especially its medial subdivision, and weaker labeling in the superior colliculus. Neuronal labeling in the medial pulvinar was likely due to a combination of monosynaptic and disynaptic transport. Previous studies showed that neurons in the medial pulvinar project to the lateral part of the amygdala (Jones and Burton 1976; Norita and Kawamura 1980; Romanski et al. 1997; Elorette et al. 2018). In addition, the involvement of disynaptic transport is possible because the medial pulvinar sends projections to the anterior part of the temporal cortex (Burton and Jones 1976; Baleydier and Morel 1992), which has extensive connections with the amygdala (Aggleton et al. 1980; Amaral et al. 1992; Cheng et al. 1997; Stefanacci and Amaral 2000). We found that the distribution of labeled neurons in the superior colliculus was sparse and nearly random. The neuronal labeling in the superior colliculus (presumably by disynaptic transport) might be underestimated because we set the survival time as short as possible to exclude trisynaptic transport. Another possibility is that the sparse labeling may actually reflect a rather weak projection from the superior colliculus to the medial pulvinar. Previous studies have shown that the projection from the superior colliculus is much stronger to the inferior and lateral subdivisions of the pulvinar than to the medial subdivision (Benevento and Fallon 1975; Benevento and Standage 1983). The sparse labeling in the superior colliculus also agrees with the physiological findings. First, the retinotopic organization is found in the lateral and inferior pulvinar (Bender 1981) but not in the medial pulvinar. Second, the response property of amygdala neurons in terms of the reference frame of SF tuning is less sophisticated than that of neurons in the temporal visual cortex (Inagaki and Fujita 2011). Limited projections from the superior colliculus might result in the simple response property of neurons in the amygdala.

These data confirm and extend findings in a recent paper (Elorette et al. 2018), which showed closely adjacent localization of the axons from the superior colliculus with the pulvinar neurons projecting to the amygdala, suggesting an anatomical route connecting the superior colliculus through the pulvinar to the amygdala. Our anatomical experiments provided direct evidence for the existence of this multisynaptic pathway, given that the rabies virus is transmitted only through synapses (Kelly and Strick 2000; Ugolini 2011). While we found that the labeled neurons were widely distributed across the layers of the superior colliculus, it is noteworthy that the labeled neurons existed in the superficial retinorecipient layers, because they might constitute the shortest pathway connecting the retina and the amygdala. Our data suggest that the labeled neurons in the superficial layers (SGS, SO) of the superior colliculus might be involved in the shortest pathway that connects the retina and the amygdala, as these layers receive direct input from retinal ganglion cells (Pollack and Hickey 1979). This is consistent with the previous finding that neurons projecting to the pulvinar are concentrated in the lower SGS and SO (Benevento and Standage 1983). Based on these anatomical results, we suggest that rapid processing of threatening facial expressions, as revealed by our electrophysiology experiments, is mediated by the colliculo–pulvino–amygdalar pathway. The existence of this pathway in the tree shrew (Casagrande et al. 1972; Luppino et al. 1988; Day-Brown et al. 2010; see Petry and Bickford 2019 for a review) also supports this view.

We found almost no labeled neurons in the LGN. This indicates that LGN neurons send signals to the amygdala through a larger number of synaptic relays than neurons in the superior colliculus. Specifically, the short survival time used in this study was insufficient to reveal the multisynaptic connections, if any, from the LGN to the amygdala. This finding suggests that the rapid response to threatening facial expression in the amygdala is independent of the geniculo–cortical pathway and instead relies on the colliculo–pulvino–amygdalar pathway.

In addition to the specific subcortical pathway mentioned above, other pathways connecting the retina and the amygdala might contribute to rapid processing. For example, labeled neurons in the deeper layers of the superior colliculus (Fig. 7F) might also be involved in rapid processing because visual response onset latency is suggested to be comparable between the superficial and deeper layers (Marino et al. 2012). We observed labeled neurons in several thalamic nuclei outside the pulvinar subdivisions, such as the nucleus limitans, suprageniculate nucleus, mediodorsal nucleus, and centre médian/parafascicular complex (Fig. 8B and C). Because the superior colliculus projects to these thalamic nuclei (Benevento and Fallon 1975; Harting et al. 1980; Benevento and Standage 1983; Elorette et al. 2018; see Basso and May 2017 for a review), labeled neurons in the superior colliculus might possibly be infected through some of these nuclei. These findings suggest that some of these thalamic nuclei outside the pulvinar may comprise other pathways for the rapid processing of facial expressions. Impaired perception of facial expressions was reported in stroke patients with lesions in the thalamus (Cheung et al. 2006). To our knowledge, however, no study has pinpointed which thalamic nuclei are important.

Another possible pathway is through the pretectum. Labeled neurons were observed in the pretectum, including the brachium of the superior colliculus (Fig. 8A) as well as more rostral regions (Fig. 8B and C). Because portions of the pretectum are retinorecipient and project to the pulvinar (Benevento et al. 1977; see Gamlin 2006 for a review), the pretectum is an additional candidate region connecting the retina and the amygdala. Moreover, visual cortical areas also potentially influence the rapid processing through projections to the superior colliculus (Harting and Updyke 2006) and/or to the medial pulvinar (Gutierrez et al. 2000; see Kaas and Lyon 2007 for a review). Further studies are required to clarify the contribution of each potential pathway to rapid processing.

Threat-related signals other than threatening facial expressions are also likely to be processed through the same subcortical pathway. For example, the latencies of responses to predators such as snakes are even shorter, on average, than those of threatening facial expressions in the pulvinar (Le et al. 2013; Nguyen et al. 2013). Additionally, objects newly associated with aversive events may also be processed through this pathway. The superior colliculus, pulvinar, and amygdala showed increased covariation for unseen fear-conditioned faces as well as unseen fearful faces in a V1-damaged patient (Morris et al. 2001). This finding was also reported in healthy subjects using a masking paradigm (Morris et al. 1999). The colliculo–pulvino–amygdalar pathway, demonstrated here, might be involved in processing diverse types of threat-related signals. Detection of threat should accompany behavioral reactions to manage subsequent potential danger. These signals might trigger not only orienting responses, but also avoidance, as shown by artificial manipulations of the activity of the superior colliculus (Hikosaka and Wurtz 1985; Sahibzada et al. 1986; DesJardin et al. 2013).

In conclusion, our physiology results, based on responses of single neurons in the amygdala and temporal visual cortex of the same individual animals, provide strong evidence for the rapid detection of threatening facial expressions by amygdala neurons independent of input via visual areas in the temporal cortex. Our anatomical data, based on multisynaptic neural tracing, support the idea that rapid threat detection is subcortically mediated by the colliculo–pulvino–amygdalar pathway. We propose a new dual-role model that incorporates local inhibitory interaction within the amygdala into the original dual-route model. The temporal asynchrony of subcortical and cortical processing, in addition to the transmission rate within the subcortical route, may be key for achieving reliable threat detection.
